# Supplementary material for: Twenty-Four-Hour Movement Behaviors and Social Functions in Neurodiverse Children: A Scoping Review
Source: Behav Sci (Basel). 2025 Apr 28;15(5):592. doi: 10.3390/bs15050592 (PMC12108895; doi:10.3390/bs15050592)
Supplement: Supplementary file 1 [file behavsci-15-00592-s001.zip › File S1. List of included studies.pdf]

Cai, K.; Yu, Q.; Herold, F.; Liu, Z.; Wang, J.; Zhu, L.; Xiong, X.; Chen, A.; Muller, P.; Kramer, A.F.; et al. Mini-Basketball Training Program Improves Social Communication and White Matter Integrity in Children with Autism. *J. Hum. Kinet.* **2020**, *10*, doi:10.3390/brainsci10110803.

Cei, A.; Ruscello, B.; Sepio, D. The role of Football in Enhancing psychosocial skills in Youth with Autism spectrum disorder. *Int. J. Sport Psychol.* **2023**, *54*, 373-388, doi:10.7352/IJSP.2023.54.373.

Coffey, C.; Sheehan, D.; Faigenbaum, A.; Healy, S.; Lloyd, R.; Kinsella, S. Changes in Behaviours Following an Integrative Exercise Intervention in Children with Autism Spectrum Disorder: The Influence of Symptom Severity. *International Journal of Disability, Development and Education* **2024**, 1-12, doi:10.1080/1034912x.2024.2317471.

Craig, S.G.; Weiss, M.D.; Hudec, K.L.; Gibbins, C. The Functional Impact of Sleep Disorders in Children With ADHD. *Journal of attention disorders* **2020**, *24*, 499-508, doi:10.1177/1087054716685840.

Dovgan, K.; Mazurek, M. Relations among activity participation, friendship, and internalizing problems in children with autism spectrum disorder. *Autism* **2019**, *23*, 750-758, doi:10.1177/1362361318775541.

Goldman, S.E.; McGrew, S.; Johnson, K.P.; Richdale, A.L.; Clemons, T.; Malow, B.A. Sleep is associated with problem behaviors in children and adolescents with Autism Spectrum Disorders. *Res. Autism Spectr. Disord.* **2011**, *5*, 1223-1229, doi:10.1016/j.rasd.2011.01.010.

Güeita-Rodríguez, J.; Ogonowska-Slodownik, A.; Morgulec-Adamowicz, N.; Martín-Prades, M.; Cuenca-Zaldívar, J.; Palacios-Ceña, D. Effects of Aquatic Therapy for Children with Autism Spectrum Disorder on Social Competence and Quality of Life: A Mixed Methods Study. *Int. J. Environ. Res. Public Health* **2021**, *18*, doi:10.3390/ijerph18063126.

Gundogmus, E.; Toplu, S.; Akyurek, G. The role of physical activity in social and behavioral skills of children with autism spectrum disorder: a case-controlled study. *International Journal of Developmental Disabilities* **2024**, 1-9, doi:10.1080/20473869.2024.2333608.

Haghighi, A.H.; Broughani, S.; Askari, R.; Shahrabadi, H.; Souza, D.; Gentil, P. Combined Physical Training Strategies Improve Physical Fitness, Behavior, and Social Skills of Autistic Children. *J. Autism Dev. Disord.* **2023**, *53*, 4271-4279, doi:10.1007/s10803-022-05731-8.

Hashemi, A.; Zamani, M.; Saadatian, A. Effect of Sensory-Motor Integration Trainings on Executive Functions and Social Interactions of Children with High Functioning Autism Disorder. *Journal of Motor Control and Learning* **2024**, *6*, doi:10.5812/jmcl-147293.

Hatipoglu Ozcan, G.; Ozer, D.F.; Pinar, S. Effects of Motor Intervention Program on Academic Skills, Motor Skills and Social Skills in Children with Autism Spectrum Disorder. *J. Autism Dev. Disord.* **2024**, doi:10.1007/s10803-024-06384-5.

Heffler, K.F.; Frome, L.R.; Garvin, B.; Bungert, L.M.; Bennett, D.S. Screen time reduction and focus on social engagement in autism spectrum disorder: A pilot study. *Pediatr. Int.* **2022**, *64*, e15343, doi:10.1111/ped.15343.

Homayounnia Firouzjah, M.; Majidi Yaeichi, N.; Hematinia, R. The Effectiveness of Sensory-Motor Integration Exercises on Social Skills and Motor Performance in Children with Autism. *J. Autism Dev. Disord.* **2024**, doi:10.1007/s10803-024-06325-2.

Howells, K.; Sivaratnam, C.; Lindor, E.; Hyde, C.; McGillivray, J.; Whitehouse, A.; Rinehart, N. Can Participation in a Community Organized Football Program Improve Social, Behavioural Functioning and Communication in Children with Autism Spectrum Disorder? A Pilot Study. *J. Autism Dev. Disord.* **2020**, *50*, 3714-3727, doi:10.1007/s10803-020-04423-5.

Kara, T.; Alpgan, Ö.; Yilmaz, S.; Akaltun, I. Sleep habits as an indicator of social competence and behaviour in pre-schoolers in the context of neurodevelopmental disorders. *Psychiatry and Clinical Psychopharmacology* **2019**, *29*, 68-75, doi:10.1080/24750573.2018.1487692.

Kaur, M.; Eigsti, I.M.; Bhat, A. Effects of a creative yoga intervention on the joint attention and social communication skills, as well as affective states of children with Autism Spectrum Disorder. *Res. Autism Spectr. Disord.* **2021**, *88*, doi:10.1016/j.rasd.2021.101860.

Keshavarzi, Z.; Bajoghli, H.; Mohamadi, M.R.; Salmanian, M.; Kirov, R.; Gerber, M.; Holsboer-Trachsler, E.; Brand, S. In a randomized case-control trial with 10-years olds suffering from attention deficit/hyperactivity disorder (ADHD) sleep and psychological functioning improved during a 12-week sleep-training program. *World J. Biol. Psychiatry* **2014**, *15*, 609-619, doi:10.3109/15622975.2014.922698.

Lee, G.T.; He, L.; Xu, S. Using Cooperative Physical Activities in Inclusive Settings to Enhance Social Interactions for Preschoolers With Autism Spectrum Disorder in China. *Journal of Positive Behavior Interventions* **2021**, *24*, 236-249, doi:10.1177/10983007211035135.

Lee, J.; Chang, S.H.; Jolin, J. Developing Social Skills of Children With Autism Spectrum Disorder for Physical Activity Using a Movement-Based Program. *Journal of Motor Learning and Development* **2021**, *9*, 95-108, doi:10.1123/jmld.2020-0017.

Liu, Z.; Herold, F.; Healy, S.; Haegele, J.; Block, M.; Ludyga, S.; Ng, J.; Gerber, M.; Hossain, M.; Taylor, A.; et al. Understanding 24-hour movement guideline adherence and links to school achievement, social-behavioural problems, and emotional functioning among children and adolescents with learning disabilities. *International Journal of Sport and Exercise Psychology* **2023**, 1-25, doi:10.1080/1612197x.2023.2288259.

Lucas, I.; Mulraney, M.; Sciberras, E. Sleep problems and daytime sleepiness in children with ADHD: Associations with social, emotional, and behavioral functioning at school, a cross-sectional study. *Behav. Sleep Med.* **2019**, *17*, 411-422.

Majorek, M.; Tuchelmann, T.; Heusser, P. Therapeutic Eurythmy-movement therapy for children with attention deficit hyperactivity disorder (ADHD): a pilot study. *Complement. Ther. Nurs. Midwifery* **2004**, *10*, 46-53, doi:10.1016/S1353-6117(03)00087-8.

Marzouki, H.; Soussi, B.; Selmi, O.; Hajji, Y.; Marsigliante, S.; Bouhlel, E.; Muscella, A.; Weiss, K.; Knechtle, B. Effects of Aquatic Training in Children with Autism Spectrum Disorder. *Biology (Basel)* **2022**, *11*, 657, doi:10.3390/biology11050657.

Memari, A.; Mirfazeli, F.; Kordi, R.; Shayestehfar, M.; Moshayedi, P.; Mansournia, M. Cognitive and social functioning are connected to physical activity behavior in children with autism spectrum disorder. *Res. Autism Spectr. Disord.* **2017**, *33*, 21-28, doi:10.1016/j.rasd.2016.10.001.

Meng, H.; Kim, Y.; Lee, K. Impact of a Service-Learning Program Using Soccer Training on the Emotional and Behavioral Problems of Children with Developmental Disabilities. *Children (Basel)* **2024**, *11*, doi:10.3390/children11040467.

Mohamed, R.I.; El-Hadidy, E.I.; Dawoud, M.; Ismaeel, M.M.I. Effect of Group Exercise Program on Social Skills and Bone Mineral Density in Children with Autism. *The Egyptian Journal of Hospital Medicine* **2024**, *96*, 2665-2671, doi:10.21608/ejhm.2024.369498.

Morris, P.O.; Hope, E.; Foulsham, T.; Mills, J.P. Exploring the use of a dance-based exergame to enhance autistic children's social communication skills in the home and school environments: a feasibility study. *International Journal of Developmental Disabilities* **2023**, 1-18, doi:10.1080/20473869.2023.2212985.

Movahedi, A.; Bahrami, F.; Marandi, S.; Abedi, A. Improvement in social dysfunction of children with autism spectrum disorder following long term Kata techniques training. *Res. Autism Spectr. Disord.* **2013**, *7*, 1054-1061, doi:10.1016/j.rasd.2013.04.012.

Najafabadi, M.G.; Sheikh, M.; Hemayattalab, R.; Memari, A.H.; Aderyani, M.R.; Hafizi, S. The effect of SPARK on social and motor skills of children with autism. *Pediatr. Neonatol.* **2018**, *59*, 481-487, doi:10.1016/j.pedneo.2017.12.005.

Narasingharao, K.; Pradhan, B.; Navaneetham, J. Efficacy of Structured Yoga Intervention for Sleep, Gastrointestinal and Behaviour Problems of ASD Children: An Exploratory Study. *J Clin Diagn Res* **2017**, *11*, VC01-VC06, doi:10.7860/JCDR/2017/25894.9502.

Ng, R.; Heinrich, K.; Hodges, E. Do You Really Lose When You Snooze? Sleep Correlates of Externalizing and Attention Problems Among Pediatric Patients with ADHD, ASD, and Comorbid Diagnosis. *Journal of Mental Health Research in Intellectual Disabilities* **2020**, *13*, 231-249, doi:10.1080/19315864.2020.1760973.

Oh, J.; McNamara, S.; Lee, Y. Exploring the impact of parent-facilitated aquatics on children with developmental disabilities - a mixed-methods study. *International Journal of Developmental Disabilities* **2024**, 1-13,

doi:10.1080/20473869.2024.2371683.
Pan, C.Y. Effects of water exercise swimming program on aquatic skills and social behaviors in children with autism spectrum disorders. *Autism* **2010**, *14*, 9-28, doi:10.1177/1362361309339496.
Pan, C.Y.; Chu, C.H.; Tsai, C.L.; Lo, S.Y.; Cheng, Y.W.; Liu, Y.J. A racket-sport intervention improves behavioral and cognitive performance in children with attention-deficit/hyperactivity disorder. *Res Dev Disabil* **2016**, *57*, 1-10, doi:10.1016/j.ridd.2016.06.009.
Phung, J.N.; Goldberg, W.A. Mixed martial arts training improves social skills and lessens problem behaviors in boys with Autism Spectrum Disorder. *Res. Autism Spectrum. Disord.* **2021**, *83*, doi:10.1016/j.rasd.2021.101758.
Qi, K.; Liu, Y.; Wang, Z.; Xiong, X.; Cai, K.; Xu, Y.; Shi, Y.; Sun, Z.; Dong, X.; Chen, A. Recreational ball games are effective in improving social communication impairments among preschoolers diagnosed with autism spectrum disorder: a multi-arm controlled study. *BMC SPORTS SCIENCE MEDICINE AND REHABILITATION* **2024**, *16*, doi:10.1186/s13102-024-00957-8.
Qiao, Z.; Sun, Z.; Cai, K.; Zhu, L.; Xiong, X.; Dong, X.; Shi, Y.; Yang, S.; Cheng, W.; Yang, Y.; et al. Effects of mini-basketball training program on social communication impairments and salience network in preschool children with autism spectrum disorder. *International Journal of Developmental Disabilities* **2024**, 1-14, doi:10.1080/20473869.2024.2394736.
Rivera, P.; Renziehausen, J.; Garcia, J.M. Effects of an 8-Week Judo Program on Behaviors in Children with Autism Spectrum Disorder: A Mixed-Methods Approach. *Child Psychiatry Hum. Dev.* **2020**, *51*, 734-741, doi:10.1007/s10578-020-00994-7.
Sahin, B.; Hosoglu, E.; Onal, B.S. The effect of sleep disturbance on social cognition in drug-naive children with attention deficit and hyperactivity disorder. *Sleep Med* **2021**, *82*, 172-178, doi:10.1016/j.sleep.2021.04.002.
Sansi, A.; Nalbant, S.; Ozer, D. Effects of an Inclusive Physical Activity Program on the Motor Skills, Social Skills and Attitudes of Students with and without Autism Spectrum Disorder. *J. Autism Dev. Disord.* **2021**, *51*, 2254-2270, doi:10.1007/s10803-020-04693-z.
Schoen, S.; Ferrari, V.; Valdez, A. It's Not Just about Bicycle Riding: Sensory-Motor, Social and Emotional Benefits for Children with and without Developmental Disabilities. *Children-Basel* **2022**, *9*, doi:10.3390/children9081224.
Shah, H.; Dani, A. A Study on Effect of Sleep Training Program in Children with ADHD: A Comparative Prospective Study. *Journal of Indian Association for Child and Adolescent Mental Health* **2023**, *19*, 370-377, doi:10.1177/09731342231184438.
Taylor, A.; Kong, C.; Zhang, Z.; Herold, F.; Ludyga, S.; Healy, S.; Gerber, M.; Cheval, B.; Pontifex, M.; Kramer, A.; et al. Associations of meeting 24-h movement behavior guidelines with cognitive difficulty and social relationships in children and adolescents with attention deficit/hyperactive disorder. *Child Adolesc. Psychiatry Ment. Health* **2023**, *17*, 42-undefined, doi:10.1186/s13034-023-

00588-w.
Toscano, C.V.A.; Ferreira, J.P.; Quinaud, R.T.; Silva, K.M.N.; Carvalho, H.M.; Gaspar, J.M. Exercise improves the social and behavioral skills of children and adolescent with autism spectrum disorders. *Front Psychiatry* **2022**, *13*, 1027799, doi:10.3389/fpsyt.2022.1027799.
Wang, J.-G.; Cai, K.-L.; Liu, Z.-M.; Herold, F.; Zou, L.; Zhu, L.-N.; Xiong, X.; Chen, A.-G. Effects of Mini-Basketball Training Program on Executive Functions and Core Symptoms among Preschool Children with Autism Spectrum Disorders. *Brain Sciences (2076-3425)* **2020**, *10*, 263.
Yang, S.; Liu, Z.; Xiong, X.; Cai, K.; Zhu, L.; Dong, X.; Wang, J.; Zhu, H.; Shi, Y.; Chen, A. Effects of Mini-Basketball Training Program on Social Communication Impairment and Executive Control Network in Preschool Children with Autism Spectrum Disorder. *Int J Environ Res Public Health* **2021**, *18*, doi:10.3390/ijerph18105132.
Yang, Y.; Chen, D.; Cai, K.; Zhu, L.; Shi, Y.; Dong, X.; Sun, Z.; Qiao, Z.; Yang, Y.; Zhang, W.; et al. Effects of mini-basketball training program on social communication impairments and regional homogeneity of brain functions in preschool children with autism spectrum disorder. *BMC sports science, medicine & rehabilitation* **2024**, *16*, 92, doi:10.1186/s13102-024-00885-7.
Zanobini, M.; Solari, S. Effectiveness of the Program "Acqua Mediatrice di Comunicazione" (Water as a Mediator of Communication) on Social Skills, Autistic Behaviors and Aquatic Skills in ASD Children. *JOURNAL OF AUTISM AND DEVELOPMENTAL DISORDERS* **2019**, *49*, 4134-4146, doi:10.1007/s10803-019-04128-4.
Zhao, M.; Chen, S. The Effects of Structured Physical Activity Program on Social Interaction and Communication for Children with Autism. *Biomed Res Int* **2018**, *2018*, 1825046, doi:10.1155/2018/1825046.
Zhao, M.; Hou, M.; Herold, F.; Chen, Y.; Werneck, A.O.; Block, M.E.; Kramer, A.F.; Taylor, A.; Cunha, P.M.; Chaput, J.P.; et al. Associations of meeting 24-hour movement behavior guidelines with social and emotional function in youth with ASD/ADHD. *J Affect Disord* **2024**, *359*, 189-195, doi:10.1016/j.jad.2024.05.086.
