# Supplementary material for: Twenty-Four-Hour Movement Behaviors and Social Functions in Neurodiverse Children: A Scoping Review
Source: Behav Sci (Basel). 2025 Apr 28;15(5):592. doi: 10.3390/bs15050592 (PMC12108895; doi:10.3390/bs15050592)
Supplement: Supplementary file 1 [file behavsci-15-00592-s001.zip › Supplementary tables.pdf]

Table S1

Keywords and search strategy to find eligible studies

Terms	Descriptors
**#1 24-hour movement behavior**	24-hour movement behavior OR 24-hour movement OR 24-hour activity OR physical activity OR exercise OR sedentary behavior OR physical inactivity OR screen time OR sleep OR insomnia OR sleepless
**#2 neurodiversity**	neurodiverse OR neurodiversity OR developmental disorders OR mental disorder OR ADHD OR attention deficit hyperactivity disorder OR ASD OR autism OR autism spectrum disorder OR developmental language disorders OR dyslexia OR dyscalculia OR aphasia OR learning difficulties
**#3 social function**	social functioning OR community functioning OR social competence OR social skill OR social interaction OR social dynamics OR interpersonal skill OR interpersonal interaction OR interpersonal dynamics
**#4 child**	child* OR kids* OR boys* OR girls* OR schoolchild* OR early-years OR preschool OR school
**Combination**	#1 AND #2 AND #3 AND #4

Table S2

Exact syntaxes used in each searched database

**Scopus**	("24-hour movement behavior" OR "24-hour movement" OR "24-hour activity" OR "physical activity" OR "exercise" OR "sedentary behavior" OR "physical inactivity" OR "screen time" OR "sleep" OR "insomnia" OR "sleepless") AND ("neurodiverse" OR "neurodiversity" OR "developmental disorders" OR "mental disorder" OR "ADHD" OR "attention deficit hyperactivity disorder" OR "ASD" OR "autism" OR "autism spectrum disorder" OR "developmental language disorders" OR "dyslexia" OR "dyscalculia" OR "aphasia" OR "learning difficulties") AND ("social functioning" OR "community functioning" OR "social competence" OR "social skill" OR "social interaction" OR "social dynamics" OR "interpersonal skill" OR "interpersonal interaction" OR "interpersonal dynamics") AND ("child*" OR "kids*" OR "boys*" OR "girls*" OR "schoolchild*" OR "early-years" OR "preschool" OR "school")
**PubMed**	((("24-hour movement behavior" OR "24-hour movement" OR "24-hour activity" OR "physical activity" OR "exercise" OR "sedentary behavior" OR "physical inactivity" OR "screen time" OR "sleep" OR "insomnia" OR "sleepless")<br>AND ("neurodiverse" OR "neurodiversity" OR "developmental disorders" OR "mental disorder" OR "ADHD" OR "attention deficit hyperactivity disorder" OR "ASD" OR "autism" OR "autism spectrum disorder" OR "developmental language disorders" OR "dyslexia" OR "dyscalculia" OR "aphasia" OR "learning difficulties"))<br>AND ("social functioning" OR "community functioning" OR "social competence" OR "social skill" OR "social interaction" OR "social dynamics" OR "interpersonal skill" OR "interpersonal interaction" OR "interpersonal dynamics"))<br>AND ("child*" OR "kids*" OR "boys*" OR "girls*" OR "schoolchild*" OR "early-years" OR "preschool" OR "school")
**EBSCO**	TI (24-hour movement behavior OR 24-hour movement OR 24-hour activity OR 24-hour physical activity OR 24-hour OR physical activity OR exercise OR sedentary behavior OR physical inactivity OR sleep duration OR sleep time OR screen time OR sleep quality OR insomnia OR sleep disturbance OR sleepless) OR AB (24-hour movement behavior OR 24-hour movement OR 24-hour activity OR 24-hour physical activity OR 24-hour OR physical activity OR exercise OR sedentary behavior OR physical inactivity OR sleep duration OR sleep time OR screen time OR sleep quality OR insomnia OR sleep disturbance OR sleepless)<br>AND<br>TI (neurodiverse OR neurodiversity OR developmental disorders OR mental disorder OR ADHD OR attention deficit hyperactivity disorder OR ASD OR autism OR autism spectrum disorder OR down syndrome OR developmental language disorders OR dyslexia OR dyscalculia OR aphasia OR learning difficulties) OR AB (neurodiverse OR neurodiversity OR developmental disorders OR mental disorder OR ADHD OR attention deficit hyperactivity disorder OR ASD OR autism OR autism spectrum disorder OR developmental language disorders OR dyslexia OR dyscalculia OR aphasia OR learning difficulties)<br>AND<br>TI (social function OR social functioning OR social skills OR social interaction OR social behavior OR social behavior OR social OR interpersonal OR interpersonal relationship OR social dynamic) OR AB (social function OR social functioning OR social skills OR social interaction OR social behavior OR social behavior OR social OR interpersonal OR interpersonal relationship OR social dynamic)<br>AND<br>TI (child OR children OR kid* OR schoolchild OR schoolchildren OR early-years OR preschool child* OR boy* OR girl* OR school) OR AB (child OR children OR kid* OR schoolchild OR schoolchildren OR early-years OR preschool child* OR boy* OR girl* OR school)
**Web of Science**	TS= (24-hour movement behavior OR 24-hour movement OR 24-hour activity OR physical activity OR exercise OR sedentary behavior OR physical inactivity OR screen time OR sleep OR insomnia OR sleepless)

	AND<br>TS= (neurodiverse OR neurodiversity OR developmental disorders OR mental disorder OR ADHD OR attention deficit hyperactivity disorder OR ASD OR autism OR autism spectrum disorder OR developmental language disorders OR dyslexia OR dyscalculia OR aphasia OR learning difficulties)<br>AND<br>TS= (Social functioning OR community functioning OR social competence) OR TS= ((social OR interpersonal) AND (skill OR interaction OR dynamics))<br>AND<br>TS= (Child* OR kid* OR boy* OR girl* OR schoolchild* OR early-years OR preschool OR school)

Table S3

Eligibility criteria and results extraction (from Peters et al., 2015)

**Review title:** Self-compassion in sport: A scoping review
**Review question:** The effect of 24-hour movement behavior on social functioning in neurodiverse children
**Inclusion criteria (PCC):**<br>*Population* – Children aged 0-18 years show at least one neurodiversity trait.<br>*Concept* – 24-hour movement behavior: It must involve patterns of 24-hour movement behavior (i.e., physical activity, sleep time, sedentary behavior), which include at least one or more types of behaviors within the 24-hour movement paradigm<br>Neurodiversity: At least one characteristic of neurodiversity (such as Autism Spectrum Disorder, Attention Deficit Hyperactivity Disorder, learning disabilities, etc.)<br>Social functioning: social functioning is used as an outcome measure<br>*Context* – Participate in physical activity or specific physical activity interventions.
**Exclusion criteria:**<br>*Non-English resources*—The confines of this particular scoping review does not allow for the time nor the resources to conduct a search outside of the primary researcher's parent language.<br>*Studies not focused on neurodiverse children*—children who do not display neurodiverse traits or have psychiatric disorders are not the focus of our target background.<br>*Research that does not involve 24-hour movement behaviour or one of its specific behaviors*—not focus on 24-hour movement behavior or on addressing only one of these behaviors.<br>*Outcome measures that are not related to social functioning*— the study specifically targets social functioning, and variables outside of this domain are not within the scope of our research.<br>*Review articles and studies that are not peer-reviewed*—gray literature such as book chapters, dissertations, conference papers.<br>*Non-empirical report*— Sources of data other than primary studies-position/opinion papers.<br>*Not full-length report*—poster, conference proceeding or abstract etc.<br>*Non-peer-reviewed research*-grey literature such as book chapters, dissertations, conference proceedings, etc.)
**Extraction fields:**<br>Author(s)<br>Year of publication<br>Name of journal<br>Source of origin<br>Country of origin<br>Aims/purpose<br>Study population/sample size<br>Methodology<br>Studies measures<br>Key overall findings that relate to the review question
